# PAirwise Sequence Comparison (PASC) and Its Application in the Classification of Filoviruses

**DOI:** 10.3390/v4081318

**Published:** 2012-08-20

**Authors:** Yiming Bao, Vyacheslav Chetvernin, Tatiana Tatusova

**Affiliations:** National Center for Biotechnology Information, National Institutes of Health, Bethesda, MD 20892, USA; Email: chetvern@ncbi.nlm.nih.gov (V.C.); tatiana@ncbi.nlm.nih.gov (T.T.)

**Keywords:** *Filoviridae*, filovirus, ICTV, International Committee on Taxonomy of Viruses, National Center for Biotechnology Information, NCBI, PAirwise Sequence Comparison, PASC, virus classification, virus taxonomy

## Abstract

PAirwise Sequence Comparison (PASC) is a tool that uses genome sequence similarity to help with virus classification. The PASC tool at NCBI uses two methods: local alignment based on BLAST and global alignment based on Needleman-Wunsch algorithm. It works for complete genomes of viruses of several families/groups, and for the family of *Filoviridae*, it currently includes 52 complete genomes available in GenBank. It has been shown that BLAST-based alignment approach works better for filoviruses, and therefore is recommended for establishing taxon demarcation criteria. When more genome sequences with high divergence become available, these demarcations will most likely become more precise. The tool can compare new genome sequences of filoviruses with the ones already in the database, and propose their taxonomic classification.

## 1. Introduction

The family *Filoviridae* belongs to the order *Mononegavirales*. Filovirus genomes are single-stranded, negative sense RNAs of about 19 kb, which encode 7 structural proteins. There are currently two genera in this family (*Marburgvirus* and *Ebolavirus*), and a third genus (“Cuevavirus”) was proposed recently to the International Committee on Taxonomy of Viruses (ICTV) [1,2]. The criteria used to separate the members of the two genera mainly include antigenic cross-reactivity, virion length, number of gene overlaps, number of protein products expressed by gene 4, and genome divergence. The genus *Ebolavirus* currently includes five species, all of which are represented by a single virus: *Bundibugyo ebolavirus* (member Bundibugyo virus), *Reston ebolavirus* (member Reston virus), *Sudan ebolavirus* (member Sudan virus), *Taï Forest ebolavirus* (member Taï Forest virus) and *Zaire ebolavirus* (member Ebola virus). The species demarcation criteria for ebolaviruses include whole genome differences, number of gene overlaps and geographic origin. The genus *Marburgvirus* and the tentative genus “Cuevavirus” each include one species, *Marburg marburgvirus* and the tentative “Lloviu cuevavirus”, respectively, and there are currently no species demarcation criteria for the two. However, whereas only one “cuevavirus”, Lloviu virus, is known, two viruses, Marburg virus and Ravn virus, have been designated members of the species *Marburg marburgvirus* [1,2,3].

PAirwise Sequence Comparison (PASC) is a tool that can help virus classification using genome sequences [4]. It calculates the percent of pairwise sequence identity for all published complete genomes within a virus family/group, and plots the frequency distribution. In many virus families/groups, there are clear peaks in percent identity that represent pairs of viruses belonging to the same species, to different species but to the same genus, or to different genera. When these peaks are well separated, the identity percentage at their boundaries can serve as one of the species/genera demarcation criteria.

In order to provide an online tool to use PASC for virus families to which this method already has been applied (e.g., families *Geminiviridae*, *Papillomoviridae*, *Picornaviridae* and *Potyviridae*), and to investigate the possibilities of applying PASC to a wider range of viral groups, the National Center for Biotechnology Information (NCBI) created a PASC resource [5] which currently covers over 50 viral families/groups. PASC can be used to evaluate whether sequence-based classification criteria can be established for these viral families/groups, and, if yes, PASC can suggest the taxonomic classification for viruses whose genome sequences are available.

Traditionally, genome identities were calculated based on pairwise global alignments (*i.e.*, sequences are aligned end-to-end) in PASC. During the development of the PASC tool at NCBI, we noticed that there were issues with the global alignment in cases where sequences have inconsistent starting position, are in different strand of the genomes, or have extremely low similarities in part of the genomes. A new method based on BLAST (*i.e.*, only regions with good alignment are used to calculate pairwise identities) was introduced to NCBI’s PASC tool, which improved the result for many families.

Here, we describe the application of PASC to the classification of filoviruses.

## 2. Results and Discussion

### 2.1. Establishing Demarcation Criteria

**Figure 1:**
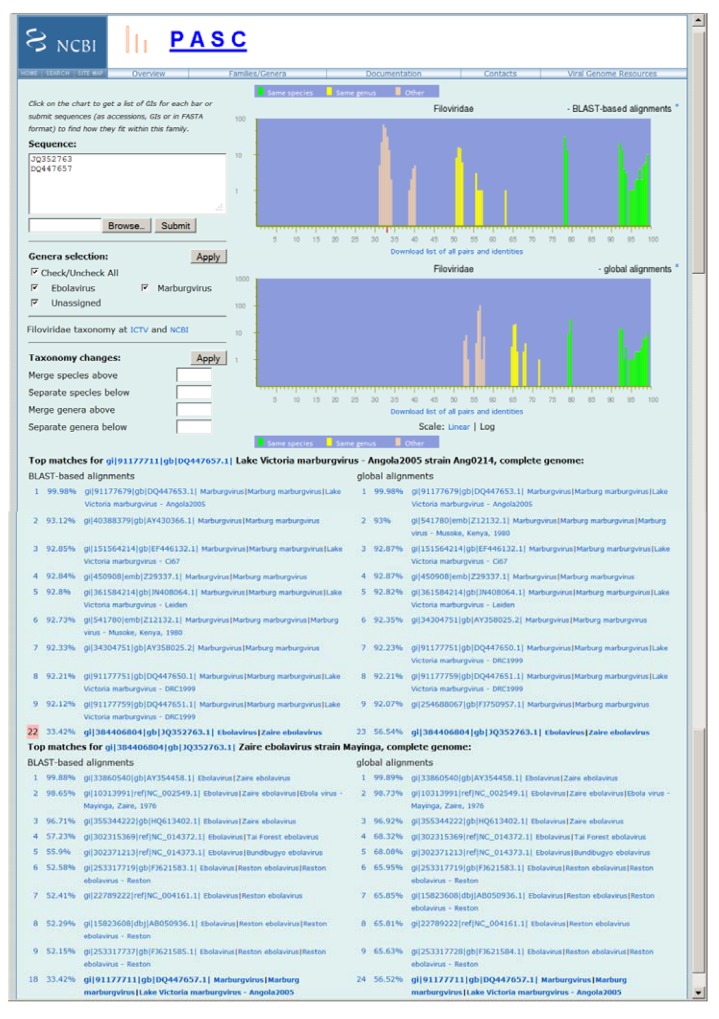
Frequency distribution of pairwise identities for the complete genome sequences of filoviruses, and the application in classifying newly sequenced viruses. The two plots represent results obtained by the BLAST-based alignments and global alignments respectively. The green, yellow and peach bars in the plots represent pairs of genomes that are assigned to the same species, different species but in the same genus, and different genera respectively in the current National Center for Biotechnology Information (NCBI) Taxonomy Database. The top matches for each input genome (JQ352763 and DQ447657) to the existing genomes in GenBank and other input genome are listed, and their pairwise identities are shown. The small red bar on the X-axis of the top plot indicates the percentage of identity of the selected pair (#22 which is highlighted). Not all virus species names listed reflect the most recent International Committee on Taxonomy of Viruses (ICTV) species names.

The PASC tool for the family of *Filoviridae* at NCBI can be accessed through [6] (a screen shot is shown in Figure 1). It currently contains 52 complete genome sequences available in GenBank. The upper right plot in Figure 1 displays the pairwise identity distribution of 28 non-redundant genomes calculated by the BLAST-based alignment, while the lower right plot shows the pairwise identity distribution of 27 non-redundant genomes by the global alignment (See Experimental Section for the alignment methods). The green, yellow and peach bars in the plots represent pairs of genomes that are assigned to the same species, different species but in the same genus, and different genera respectively in the current NCBI Taxonomy Database. PASC works best when the bars of different colors group together and are well separated, which is the case in both plots in Figure 1. The results clearly indicate that (a) sequence-based classification agrees with the current ICTV taxonomy for the family *Filoviridae*; (b) the taxonomic assignments of the filovirus genome sequences in GenBank are accurate; (c) species and genera demarcations can be set for the family.

**Figure 2:**
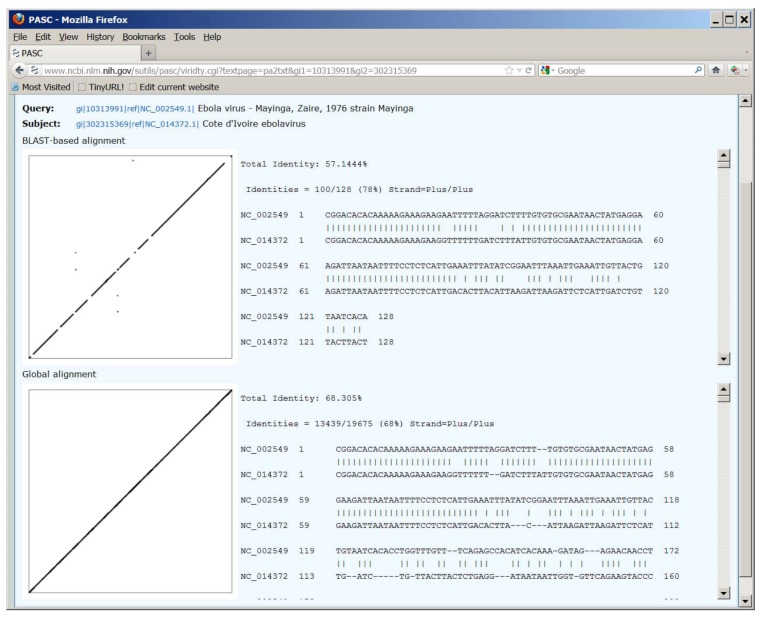
Dot matrix and text views of pairwise alignment between genome sequences of Ebola virus (NC_002549) and Taï Forest virus (NC_014372), using the BLAST-based and global alignment methods. Not all virus species names listed reflect the most recent ICTV species names.

It can be seen from Figure 1 that the yellow and peach peaks are different in the two distribution plots. For the BLAST-based alignment plot, the yellow peaks are located between 50% and 64%, and the peach ones are between 30% and 41%; while in the global-alignment plot, they are between 64% and 72%, and between 52% and 58%, respectively. Originally, the Needleman–Wunsch global‑alignment method [7] was used to calculate the genome identities in PASC [4]. It was later observed that this method was not optimized for some virus families [8]. For filoviruses, this is illustrated on Figure 2, where the dot matrix and text alignments between the genomes of Ebola virus (NC_002549) and Taï Forest virus (NC_014372) are shown (Similar alignments can be obtained by clicking the percentage of identities for the pairs of interest such as those listed in Figure 1). The identity between the two genomes calculated using the BLAST-based and global alignment is 57% and 68% respectively, neither of which matches the 63% reported by Towner *et al.* [9]. The BLAST-based method calculates percent identity only in the regions of alignment that in the case of filoviruses correspond to the 7 filoviral genes (see the dot matrix view at the upper section in Figure 2). This method essentially resembles the strategy used to classify bacteriophages based on protein sequence similarities [10,11]. The global alignment method forces alignment over the whole length of the sequence (see the dot matrix view at the lower section in Figure 2). Since, on average, any two random nucleotide sequences of the same size will have an identity of 25%, the identities obtained by the global alignment method are usually inflated, especially for genomes with high divergence. We therefore believe the BLAST‑based alignment result represents the true relationship among genome sequences of filoviruses, and should be used to establish taxa demarcation criteria, which are between 64% and 77% for species, and between 41% and 50% for genera. These demarcation criteria better match those set up by the ICTV *Filoviridae* Study Group, which are currently set as 70% for species and 50% for genera [1]. The demarcation criteria suggested by PASC are in a range rather than precise percentages as in many other viral groups, as the filovirus genomes sequenced so far are not very diverse. When more genome sequences become available, these demarcations will most likely change to narrower ranges or even to a single percentage.

It is important to keep in mind that the demarcations obtained by the BLAST-based alignment in PASC are different from those determined by other algorithms using different datasets and/or different genome regions, and therefore can only be used in the PASC system at NCBI. In another word, one cannot apply the demarcations discussed here to sequence identities calculated by other algorithms such as ClustalX, or using a single gene rather than the whole genome. 

### 2.2. Classification of Lloviu Virus

Lloviu virus was recently discovered and proposed to represent a new species in a new genus, based on the differences in its genome sequence and gene products compared to those of existing genera [12]. Its genome shows higher similarity to genomes of ebolaviruses than to marburgvirus genomes. In the PASC result, the peach color peak between 38% and 40% in the BLAST-based alignment method (upper right plot in Figure 1) represents pairs of Lloviu virus and ebolavirus genomes. If the classification was solely determined by PASC, the genera demarcation could also be set to between 35% and 38%, which would put Lloviu virus in the genus *Ebolavirus*. This demonstrates that the determination of demarcation by PASC can be arbitrary, and should not be used as the only criterion for virus classification. Other biological properties have to be taken into consideration.

### 2.3. Sub-Species Grouping of Marburgvirus

Although only one species is currently recognized in the genus *Marburgvirus*, five major lineages can be found in the phylogenetic analysis of all genomes under the species [1]. The four more closely related lineages constitute Marburg virus (MARV), and the distant lineage constitutes Ravn virus (RAVV).

**Figure 3:**
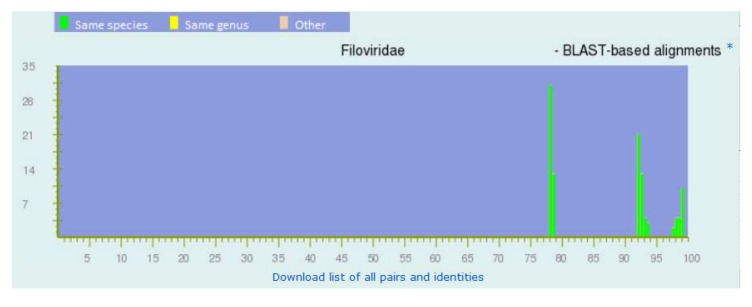
Frequency distribution of pairwise identities from the complete genome sequences of marburgviruses. The three peaks from right to left represent the pairs of viruses within each of the five major lineages of marburgviruses, between the four lineages of Marburg virus (MARV), and between Ravn virus (RAVV) and MARV.

The PASC result for the species *Marburg marburgvirus* (Figure 3) supports this division. In Figure 3, the peak above 97% represents the pairs of viruses within each of the five major lineages; the peak between 91% and 94% represents viruses between the four lineages of MARV; and the peak below 79% represents viruses between RAVV and MARV. Since these peaks are well-separated, PASC can be used to suggest sub-species classifications of newly sequenced marburgviruses.

Similar sub-species patterns are not observed in PASC for ebolaviruses, and the identities of members within each ebolavirus species are above 94% (data not shown), suggesting that intra-species variations of ebolaviruses are less than those of marburgviruses, at least when based on currently available genomes.

### 2.4. Classifying Newly Sequenced Viruses

The use of PASC for classification of viruses with newly sequenced genomes is demonstrated in Figure 1. The accession numbers of two filovirus genomes, DQ447657 (Marburg virus—Angola2005 isolate Ang0214) and JQ352763 (Ebola virus isolate Mayinga), were entered into the “Sequence” box of the PASC page for the *Filoviridae* family, and compared with existing filovirus genomes and with each other.

The nine sequences most similar to DQ447657 are listed in Figure 1, together with their pairwise identities. The most similar was DQ447653 (Marburg virus—Angola2005 isolate Ang1379c), with a sequence identity of 99.98% by the BLAST-based alignment method. Since this is in the region consisting of green bars, it suggests that DQ447657 and DQ447653 belong to the same species (*i.e.*, that DQ447657 represents a variant of marburgviruses).

Similarly, a sequence identity of 99.88% between JQ352763 and its closest neighbor, AY354458 (Ebola virus variant Kikwit) suggests that JQ352763 represents a variant of ebolaviruses.

The identity of 33.42% between the two input sequences DQ447657 and JQ352763 is also reported by PASC (see the pair number 22 highlighted in pink background when the number is clicked). Such a report is very useful in determining the relationships among genomes of novel species—if the new genomes are similar enough to each other (*i.e.*, their identity is in the green peaks), they represent the same new species; otherwise, they represent different new species.

## 3. Experimental Section

### 3.1. Source of Genome Sequences and Taxonomy Information

Complete genome sequences of viruses in the family *Filoviridae* were retrieved from the NCBI viral genome collection [13], which contains both reference sequences and other genomes for all species. There are currently 52 complete genome sequences in the database (as of July, 2012). The data set is updated daily to reflect sequence and/or taxonomy changes and to include new genomes released in GenBank.

### 3.2. Pairwise Genome Alignment and Identity Calculation

Pairwise global alignment of the genomes were performed and their identities were calculated as described before [4].

For BLAST-based alignment, two sets of BLAST [14] searches were performed on each pair of genome sequences. In the first set, the nucleotide sequence of one genome was translated to protein sequence in all six frames and searched against the nucleotide sequence of the other genome using tblastn (tblastn searches translated nucleotide database using a protein query). The amino acid alignments were then converted back to nucleotide alignments. In the second set, two genome sequences were compared using blastn (nucleotide to nucleotide alignment), followed by the creation of a consistent and non-redundant set by trimming the overlapping hits from both sets. Pairwise identity was calculated as the total number of identical bases in the local hits divided by the longest sequence length.

### 3.3. Removal of Redundant Sequences

For optimization purposes, sequences with identities higher than 99.5% were represented by one sequence in the dataset, excluding redundant sets. As a result, 27 and 28 non-redundant genomes in the family of *Filoviridae* were used in the global and BLAST-based alignment method, respectively. The numbers of non-redundant genomes are different for the two methods, because the global alignment method results in higher identities (see discussion in Section 2.1) therefore more redundant sequences.

### 3.4. Identity Distribution Plot

The identity distribution chart was plotted based on pairwise alignments computed between all non‑redundant genomes. The pair is represented in green if both genomes belong to the same species according to their assignment in NCBI’s taxonomy database; in yellow if the two genomes belong to different species within the same genus; and in peach if they belong to different genera. Both linear and log scales are available for the Y-axis (number of pairs).

### 3.5. Compare External Genomes against Existing Ones

To compare external (or new) genomes against existing sequences in the database, a user may specify the query genomes in the “Sequence:” box using either their GenBank Accession/GI numbers, entering the raw data in FASTA format, or uploading a sequence file after clicking the “Browse” button. Up to 25 sequences can be added in one submission. After sequences are submitted, PASC will compute pairwise identities between user-provided genomes and the existing genome sequences within the family. PASC produces a list of pairwise identities, from the highest to the lowest, between this input genome and (1) the rest of input genomes (if there are more than one), and (2) 5 to 10 closest matches to existing genomes within the family. The identity distribution chart will depict the currently selected genome with a different color. The user can click on each genome's number to make it current, or can click the identity to see details of the alignment.

## 4. Conclusions

The PASC tool was created at NCBI using two pairwise alignment methods on all available complete genome sequences in public databases, and is available for the family *Filoviridae*. The BLAST-based alignment result better represents the true similarities among genome sequences of filoviruses, and is recommended for establishing the demarcation criteria, which are between 64% and 77% for species, and between 41% and 50% for genera. When more genome sequences with high divergence become available, these demarcations will most likely change to narrower ranges or even to a precise percentage. These demarcations are specifically associated with the method used here therefore should not be used as references for identities calculated using other algorithms. This tool can compare new genome sequences of filoviruses with existing ones in the database, and propose their taxonomic classification.
